# Risk factors for severe COVID-19 in people with cystic fibrosis: A systematic review

**DOI:** 10.3389/fped.2022.958658

**Published:** 2022-08-08

**Authors:** Vito Terlizzi, Marco Antonio Motisi, Roberta Pellegrino, Rita Padoan, Elena Chiappini

**Affiliations:** ^1^Department of Paediatric Medicine, Cystic Fibrosis Centre, Anna Meyer Children’s University Hospital, Florence, Italy; ^2^Paediatrics Resident, Department of Health Sciences, Anna Meyer Children’s University Hospital, University of Florence, Florence, Italy; ^3^Department of Paediatrics, Cystic Fibrosis Regional Support Centre, ASST Spedali Civili di Brescia, University of Brescia, Brescia, Italy; ^4^Infectious Diseases Unit, Department of Health Sciences, Anna Meyer Children’s University Hospital, University of Florence, Florence, Italy

**Keywords:** COVID-19, cystic fibrosis, risk factors, SARS-CoV-2, CFTR modulators

## Abstract

**Background:**

People with cystic fibrosis (CF) are considered a clinically fragile population with an intrinsic higher risk of developing severe COVID-19, though a certain variability in terms of outcomes and hospitalization has been noticed.

**Aim:**

To highlight the main risk factors for severe COVID-19 in patients with CF.

**Methods:**

A systematic review of the current literature was conducted through PubMed and EMBASE databases. English-written articles reporting clinical data on CF subjects with SARS-CoV2 infection were included and analyzed. Selected reports were evaluated for adherence to STROBE recommendations.

**Results:**

After the selection phase, 9 observational studies were included, 5 of which reported data from CF Registry Global Harmonization Group. The hospitalization rate ranged from 18.2 to 58.1%. The main risk factors for severe outcome were as follows: FEV1 < 70%p, CF-related diabetes, age > 40 years, pancreatic insufficiency, underweight, previous transplant, azithromycin use. Use of dornase alfa was associated with decreased risk for severe disease, while there was insufficient evidence to establish the role of inhaled steroids or *CFTR* modulators. No solid data regarding specific SARS-CoV-2 therapies in patients with CF emerged.

**Conclusion:**

Most people with CF experience a mild course of SARS-CoV-2 infection, nevertheless subgroups with higher risk of severe outcome emerged. Maintenance therapies for CF overall did not show a clear preventive effect against severe outcomes, although dornase alfa seems to give some protection. Due to the current lack of data on specific COVID-19 therapies and immunization in patients with CF, further studies are needed to establish their impact in this population.

## Introduction

Cystic fibrosis (CF) is the most common life-threatening autosomal recessive disease in Caucasian populations, caused by variants in the *CF transmembrane conductance regulator (CFTR)* gene, in chromosome 7, coding for an ion channel protein.

CFTR variants impact on the production, trafficking, functioning or stability of the protein, mainly leading to reduced secretion of chloride, a marked absorption of sodium and water, through the epithelium, resulting in the formation of thickened secretions in organs such as lung or pancreas (1, 2).

Typically, CF is characterized by elevated sweat chloride levels, obstructive lung disease, chronic bacterial infections of lower airways and sinuses, bronchiectasis and male infertility due to obstructive azoospermia (1, 2). Most patients with CF (pwCF) have pancreatic insufficiency (PI), although 10–15% have normal exocrine pancreatic function and frequently show a milder clinical picture (3).

Progress in the development of new drugs, as *CFTR* modulators targeting the basic molecular defect in the majority of pwCF (4), has been substantial over the past decade, significantly improving clinical outcomes. It is expected to further improve patient survival in the coming years (5).

Severe acute respiratory syndrome coronavirus-2 (SARS-CoV-2), associated with the ongoing coronavirus disease 2019 (COVID-19) pandemic, has had a huge impact on world population. The presence of co-morbidities, such as CF, has been identified as a risk factor for severe disease (6, 7). The incidence is higher in people with CF versus the age-matched general population and significantly higher rates of admission to hospital and higher rates of intensive care have been reported, especially in patients receiving an organ transplant (7, 8). Mild illness was reported in CF children who did not have pre-existing severe lung disease (9).

Recently, with the implementation of the vaccine campaign, distancing measures were relaxed, and new variants of SARS-CoV-2 started to circulate. Some of the new variants showed increased transmissibility and uncertain consequences on the protection given by previous infections and vaccines (10–12).

Since the onset of the COVID-19 pandemic, CF communities, both national and international, have provided information on the clinical situation and outcomes of pwCF who have experienced SARS-CoV-2 infection.

Therefore, a review of published papers regarding COVID-19 in pwCF has been conducted with the aim of highlighting the main risk factors for severe COVID-19 disease in this population.

## Methods

Article research was conducted through MEDLINE/PubMed and EMBASE databases, including articles published from the 1st of January 2020 to the 6th of November 2021. References of all relevant articles were also evaluated, and pertinent articles were included. The search strings were as follows: “(SARS-CoV-2[Title/Abstract] OR COVID [Title/Abstract] OR COVID-19[Title/Abstract] OR SARS-CoV2[Title/Abstract]) AND (Cystic Fibrosis [Title/Abstract] OR mucoviscidosis [Title/Abstract])” for MEDLINE database and “(“SARS-CoV-2”:ab,ti OR COVID:ab,ti OR COVID-19:ab,ti OR “SARS-CoV-2”:ab,ti) AND (“cystic fibrosis”:ab,ti OR mucoviscidosis:ab,ti)” for EMBASE database.

### Inclusion and exclusion criteria

The research was restricted to the English language. Articles reporting clinical data on pwCF with COVID-19 both in pediatric and adult population were included. Articles reviews, case reports, commentaries, editorials, letters to the editor and pre-print records were excluded.

### Data extraction

Duplicate publications were removed, then two authors separately (RP and MAM) checked the titles and abstracts, and removed irrelevant studies according to the inclusion and exclusion criteria. Relevant articles from the bibliographic references of the selected studies were also considered, and an additional review of the literature was performed prior to final drafting. From each study, data regarding patients’ characteristics were extracted.

### Quality assessment

**FIGURE 1:**
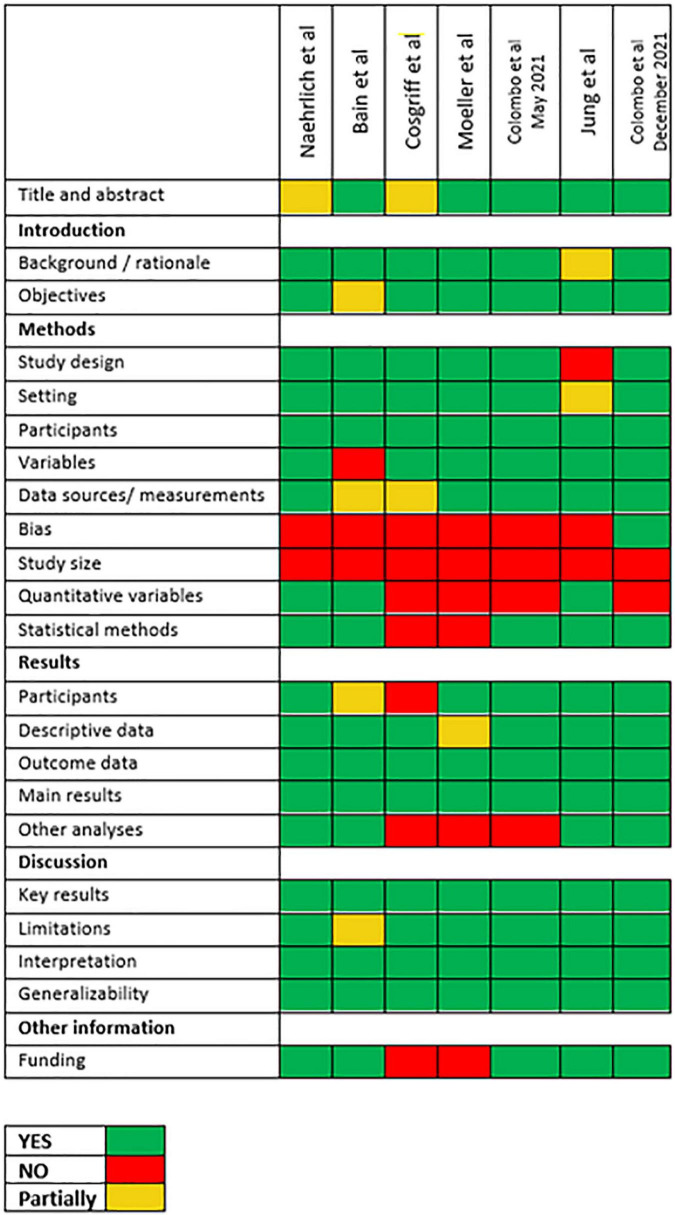
Adherence of selected studies to STROBE recommendations.

Observational studies were evaluated for adherence to Strengthening the Reporting of Observational Studies in Epidemiology (STROBE) recommendations, as reported in Figure 1.

### Ethics

Ethics approval was not required.

## Results

**FIGURE 2:**
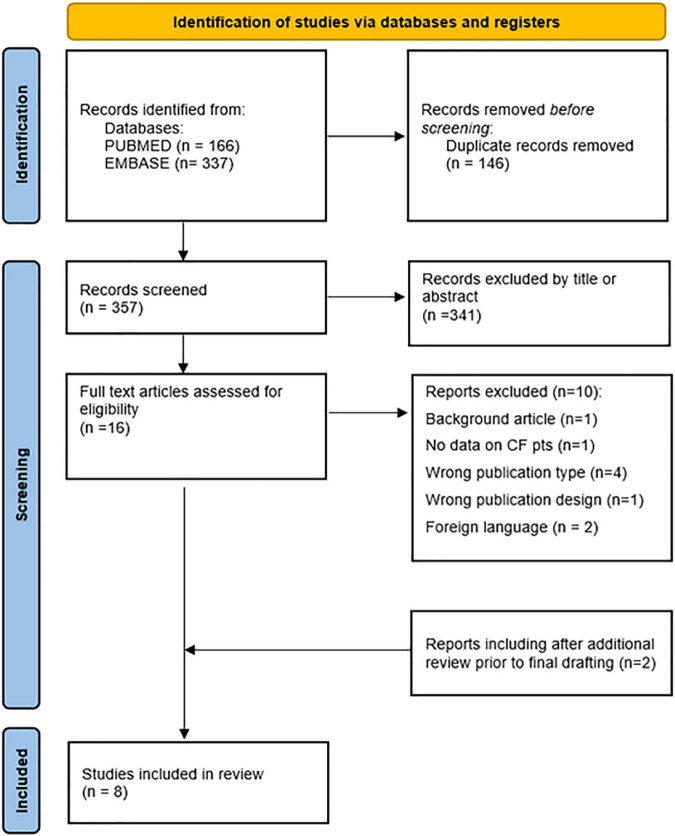
PRISMA flow diagram for literature research and data extraction.

A total of 503 publications were retrieved based on the search strings. After removing duplicates, 357 articles were retained. 341 articles were excluded by title or abstract. Of the remaining 16 articles, 10 were excluded due to not meeting inclusion criteria. Six observational studies were included. Two reports were included after additional review prior to final drafting. Literature selection process is shown in Figure 2, while characteristics and findings of selected studies were summarized in Supplementary Table 1.

Five of the selected studies included patients from the CF Registry Global Harmonization Group, an international database in which characteristics of people with CF and SARS-CoV-2 infection are gathered to evaluate the epidemiology, clinical features and prognostic factors (6–9, 13); from the global registry encompasses the European CF Society Patients’ Registry (ECFSPR) and from the national registries of Australia, United States, Canada, South Africa and South America. Two of these five studies collected patients’ information only from the European Registry (7, 8).

Among these reports, one of the earliest studies (April 2020) included 40 patients from 8 countries (median age 33 years, IQR 15–57 years) (13). Twenty-five (63%) out of 40 received antibiotic therapy and 13 (33%) out of 40 required supplemental oxygen. Of the 4 patients who needed intensive cares, 3 were organ transplant recipients. The same authors conducted a later analysis on 181 patients from 19 countries, including 149 non-transplanted and 32 organ transplanted people (6). In this study, people who underwent previous organ transplant were more likely to be hospitalized compared with non-transplanted (74% vs. 46% respectively; *p* = 0.009); 11 people were admitted to intensive care (7 post-transplant) and 7 died (3 post-transplant). In the non-transplanted cohort, low forced expiratory volume in 1 s percent predicted (FEV_1_pp) was also correlated with higher hospitalization risk (68, 71, and 28% in the FEV_1_ < 40%p, FEV_1_ 40–70% p and FEV_1_ > 70%p cohorts, respectively; *p* < 0.001).

In Europe, Jung et al. described a large cohort of 828 patients from 26 countries contributing to the ECFSPR, expanding a previous report of the same authors including 130 patients (7, 8). The previous paper, focused on the comparison with the general population, had shown a higher incidence of SARS-CoV-2 infection and hospitalization in pwCF than in the general age-matched population. In this second study, different clinical features emerged as risk factors for hospital admission: mainly FEV_1_ < 70%p (OR 8.1, *p* < 0.001), CF-related diabetes (CFRD) (OR 2.6, *p* < 0.001), age > 40 years (OR 2.4, p < 0.001), PI (OR 1.8, *p* = 0.005), underweight (OR 3.8, *p* < 0.001), previous lung transplant (OR 3.9, *p* < 0.001), azithromycin use (OR 2.7, *p* < 0.001). In addition, *CFTR* modulator use was found to be a possible protective factor against hospital admission (OR 0.7, CI 95% 0.5 – 1, *p* = 0.058). These findings overall confirmed those reported in the previous cohort of 130 patients (7, 8).

Similar results were described in an Italian multicenter study that included 236 adults and children (11). PI was found to be one of the main risk factors for severe COVID-19 (OR 4.04, *p* = 0.005). After adjusting for age and pancreatic status, FEV_1_ < 40%p (OR 4.54, *p* = 0.005), previous oxygen therapy (OR 12.27, *p* < 0.001), organ transplant (OR 7.31, *p* < 0.001), liver disease (OR 3.67, *p* < 0.001), being underweight (OR 2.92, *p* = 0.028), CFRD (OR 2.67, *p* = 0.013), azithromycin chronic therapy (OR 2.58, *p* = 0.009) emerged as the main risk factors for severe disease course. Use of dornase alfa was associated with a decreased risk for severe disease (OR 0.34, *p* = 0.026), while there was insufficient evidence to establish the role of inhaled steroids or *CFTR* modulators.

In the pediatric CF population, a multinational cohort study describes 105 children with SARS-CoV-2 infection (9). Of the 82 children for whom information on care setting were available, 24 (29%) were hospitalized, and one child needed intensive cares. Lung function and body mass index (BMI) Z-score were both found to be significantly lower in hospitalized children, compared to non-hospitalized ones (median FEV_1_ 73% predicted vs 97% predicted, respectively, *p* = 0.002; median BMI −0.55 vs 0.32 respectively, *p* = 0.015).

Given the overall paucity of data, the overlap of patients in different studies, recruitment from the same databases, and the significant heterogeneity among studies, we could not elaborate a meta-analysis in this review.

Summary of results are reported in Table 1.

**TABLE 1:** Risk and protective factors for Hospital admission in COVID-19 pwCF.

Risk factors	Authors (nr. Ref.)	Nr. pwCF studied
FEV_1_ pp < 70	Jung et al. (8); McClenagh et al. (6); Bain et al. (9)	828; 181; 105
FEV_1_ pp < 40	McClenagh et al. (6); Colombo et al. (11)	181; 236;
CFRD	Jung et al. (8); Colombo et al. (11);	828;236
Age > 40	Jung et al. (8)	828
Pancreatic insufficiency	Jung et al. (8); Colombo et al. (11);	828;236
Underweight	Jung et al. (8); Colombo et al. (11); Bain et al. (9)	828;236; 105
Organ transplant	McClenagh et al. (6);	181
Lung transplant	Jung et al. (8); Colombo et al. (11);	828;236
Azithromicyn use	Jung et al. (8); Colombo et al. (11);	828;236
Liver disease	Colombo et al. (11)	236
Previous Oxygen therapy	Colombo et al. (11)	236
***Protective factors***		
*CFTR modulators*	Jung et al. (8)	828
*Dornase alfa*	Colombo et al. (11)	236

## Discussion

### What are the main risk factors for severe COVID-19 in people with cystic fibrosis?

The presence of a severely deteriorated lung function before the SARS-CoV-2 infection was found to be one of the most relevant predictors of severe disease.

Jung et al. in a multicenter European cohort on 828 patients from the ECFSPR, (8) reported that patients with FEV_1_ < 40%p had a significantly increased risk of needing hospitalization; these data were consistent with previous findings of the same authors in the early pandemic period (7). People with moderate FEV_1_ impairment (40–70% of predicted) also were found to have an increased risk for hospital admission, although they were less likely to need supplemental oxygen (8). The prospective multicenter study of Colombo et al., including 236 patients with CF and COVID-19 also found a significant correlation between severely impaired lung function and risk of hospital admission (11).

In Jung’s cohort, patients who underwent lung transplantation were > 2-fold more likely to need hospitalization and respiratory support compared to non-transplanted patients, and also were > 6-fold more likely to be admitted in an ICU or die (8).

In Colombo’s study, organ transplantation status and need for oxygen therapy before SARS-CoV-2 infection has been identified as the major risk factors for hospitalization (11). The prognostic role of organ transplantation, partially explained by the use for immunosuppressants, had also been described in earlier studies, in which organ recipients were found to have higher hospitalization rates, need for ICU and risk of death (6, 7, 14).

Patients with PI constitute one of the CF cohorts with significant higher risk for hospitalization (8, 9, 11, 14). In the aforementioned study of Colombo et al., all the 6 patients who died had PI (11), and in the international multicenter study of Bain et al. on 105 children, all patients who needed hospital admission (*n* = 24) had PI (9).

Pancreatic insufficiency is conferred by variants of the *CFTR* gene both leading to absence or marked reduction in *CFTR* protein activity, thus ultimately resulting in severe clinical outcomes such as advanced lung disease, malnutrition, or the presence of CFRD, factors associated with the risk of severe SARS-CoV-2 infection.

Really, CFRD was found to dramatically increase chance of hospitalization and need for supplemental oxygen (7, 8, 11). Being underweight, although with slight definition differences between studies, was also found to be a significant risk factor for hospital admission and need for oxygen therapy (8, 9, 11).

Male sex is considered an independent risk factor for severe COVID-19 in the general population, but its role is uncertain among people with CF. In the multinational cohort of 181 patients described by McClenaghan et al., male sex did not appear to increase the risk of hospitalization, with 38/76 females (50%) and 28/65 males (43%) needing to be admitted (6). However, in the post-transplant cohort (*n* = 32), male patients had an higher chance to be hospitalized and need for ICU. Likewise, two other studies found no significant differences between males and females in COVID-19 outcome among pwCF; (8, 11) since females usually have a more severe clinical course of CF, (15) it has been postulated that increased risk for severe COVID-19 in males could be compensated by a worse general outcome of CF in females (8).

Chronic airways colonization by *Pseudomonas aeruginosa* is a common finding among patients with CF (1, 2). In pwCF with COVID-19, *Pseudomonas aeruginosa* chronic infection has been associated with higher risk of hospitalization and need for oxygen therapy (6, 8). Moreover, azithromycin use prior to SARS-CoV-2 infection is also suspected to be a risk factor for severe COVID-19, possibly because it is a marker of underlying *Pseudomonas aeruginosa* colonization and poor lung function (8, 11). However, since there are no studies comparing COVID-19 outcome related to azithromycin intake, the prognostic role of chronic azithromycin use remains unclear. Moreover, patients who were in chronic therapy with inhaled antibiotics were also found to be at increased risk for severe COVID-19, with higher risk of hospitalization, oxygen therapy or respiratory support (8). Regarding other colonizing bacteria, only *Achromobacter spp* has been linked to significantly increased risk for hospitalization, while patients with chronic infection by other observed species (*Stenotrophomonas maltophila*, methicillin-resistant *Staphylococcus aureus*, *Burkholderia cepacia* complex) have not shown relevantly higher risk (8).

The role of underlying pathogenic *CFTR* variants has also been investigated, comparing F508del variant (homozygous and heterozygous) with other *CFTR* variants: no statistically relevant increase in risk for severe COVID-19 was found for any genetic subgroup (7, 8, 11). Nevertheless, we cannot exclude as a confounding factor on the effects of SARS-CoV-2 infection in CF, the more frequent use of *CFTR* modulators in patients with at least one F508del, being possible that these drugs might exhibit a preemptive effect in this subgroup.

However, Jung et al. reported more frequent symptoms among patients with at least one F508del variant when compared to those with no F508del variant in the European CF population (8).

### What are the main protective factors against severe COVID-19 in people with cystic fibrosis?

SARS-CoV-2 infection usually has a milder course in the pediatric age, both among people with CF and in the general population, with overall lower risk for hospitalization, need for supplemental oxygen, ICU admission and death (6, 8, 11, 13). In CF, this could also be explained by the progressive nature of the disease, which leads to ongoing worsening of the respiratory function.

Regarding CF maintenance therapy, available literature reports conflicting conclusions. With regards to therapies with *CFTR* modulators, different rates of their use are reported: from 12.5% in a limited number of Italian patients in the Colombo’s paper (11) to 60% in the pediatric population from Europe, Russia United States, and South America described by Bain et al. (9).

The European pediatric population received *CFTR* modulators (any) in 35.6% of cases, (16) so even if these therapies seem to have a protective role, lowering the risk of hospitalization in children as in the study of Bain et al. (9), on the other hand, the highest frequency in infected children questions a protective role against infection itself in this age group.

Registries data rate of *CFTR* modulators (any) prescribed in adult population vary between 34.4% in Europe and 86.1% in United States (17). However, in studies from ECFSPR (7, 8) and Global Harmonization Group (6, 13) data are reported for the whole population and varied between 24.6% (7) and 43% (6), suggesting anyway an overall low rate of patients on *CFTR* modulator infected by SARS-CoV-2. On the contrary, data from CFF United States Registry report a 74.5% of a *CFTR* modulator prescription among 993 subjects of the COVID-19 2020 cohort (18).

The use of *CFTR* modulators, to improve the function of the *CFTR* protein or facilitate its positioning in the cell membranes, has been investigated as a potential protective factor. *CFTR* modulators lowered the risk of hospitalization in children (9), while in two other studies, on both pediatric and adult patients, these drugs did not show a significant protection against hospitalization, need for oxygen or respiratory support, or ICU admission (8, 11). Also, no difference was found between ivacaftor alone for gating *CFTR* variants and elexacaftor/tezacaftor/ivacaftor (11), both defined as highly effective modulator treatments. In one study, 60 patients receiving therapy with dornase alfa showed a decreased risk for severe COVID-19, possibly due to disruption of neutrophil extracellular nets, which are involved in COVID-19 pathogenesis (11). Finally, there were no evidence that chronic therapy with inhaled steroids before SARS-CoV-2 infection could lower the risk for severe COVID-19 (8, 11).

In the present review, the protective role of SARS-CoV-2 immunizations in pwCF could not be established, since most of the data has been collected from patients infected in the first wave of the pandemic. Only one study included patients who had been infected after the start of the immunization campaign, and a progressive decrease of COVID-19 cases among people with CF was reported from mid-March 2021 to June 2021, although solid conclusions on protective effects against risk of infection or severe disease could not be drawn (11).

### What are the possible therapies for COVID-19 in people with cystic fibrosis?

Most symptomatic patients received oral or intravenous antibiotic therapy as a standard treatment for any CF exacerbation (9, 11, 13, 19). The use of oral and inhaled corticosteroids as therapy for COVID-19 in the early periods of the pandemic has been described, but to date no studies have been conducted evaluating their use in CF cohorts.

Since most of the reports collected data from infected patients during the first wave of the pandemic, treatment information included data on use of drugs with supposed antiviral action (hydroxychloroquine, azithromycin, ritonavir/lopinavir, darunavir/cobicistat) (9, 14, 19). However, these are no longer used due to lack of evidence of efficacy or because of safety issues. The only two antiviral drugs approved in Europe for mild-moderate COVID-19 at high risk of developing severe or critical disease are molnupiravir and remdesivir, but to our knowledge there are no studies that evaluates efficacy and safety of these medications in pwCF. Monoclonal antibodies have shown to reduce viral load and incidence of severe COVID-19 in high-risk patients when administered within the first 10 days from symptoms onset. However, no solid data regarding administration of monoclonal antibodies in pwCF emerged, so further studies are required to assess their impact in this population.

### Limitations

This review has some limitations. COVID-19 pandemic is a rapidly changing situation, with ongoing developing of new therapeutic/prophylactic strategies that could not be taken into account in our work, and evolutions of new viral variants which can potentially define a whole new clinical context. Since most of patients’ information from different studies was retrieved from the same data source, i.e., from the Cystic Fibrosis Registry Global Harmonization Group, (11–14, 18) overlapping between patient datasets must be taken into account when interpreting the result of our review. This fact, along with the overall scarcity and heterogeneity of data, precluded us from making a meta-analysis of the selected studies.

## Conclusion

Even though most people with CF experience a mild course of SARS-CoV-2 infection, there are different subgroups with higher risk of severe outcome, especially patients with previous need for oxygen therapy, severe lung function impairment, those with PI, CFRD and those who underwent organ transplantation. Younger patients usually have a less severe disease. Maintenance therapies for CF overall did not show a clear preventive effect against severe outcomes, although dornase alfa seems to give some protection.

Further studies are needed to establish the role of the newest available therapies in pwCF with COVID-19 and the efficacy of immunization strategies.

## Data availability statement

The original contributions presented in the study are included in the article/Supplementary material, further inquiries can be directed to the corresponding author.

## Author contributions

All authors contributed equally to the contents of this manuscript.
