# Supplementary material for: Risk factors for severe COVID-19 in people with cystic fibrosis: A systematic review
Source: Front Pediatr. 2022 Aug 8;10:958658. doi: 10.3389/fped.2022.958658 (PMC9393295; doi:10.3389/fped.2022.958658)
Supplement: Supplementary file 1 [file Table_1.DOCX]

| Author | Country | Study design | Patient (n) | Age | Sex | Diagnosis | CF mutation | Diabetes | *P.aeruginosa* | *B. cepacia* | Pancreatic insufficiency | CF therapy | Transplanted | ICU | Deaths | Hospitalized | Treatment for COVID19 | Notes |
| --- | --- | --- | --- | --- | --- | --- | --- | --- | --- | --- | --- | --- | --- | --- | --- | --- | --- | --- |
| Naehrlich et al (7) | Europe  ECFSPR | Prospective cohort study | 130 | 0-11 15 (11.5%)  12-17 24 (18.5%)  18-29 37 (28.5%) 30-49 49 (37.7%) 50+  5 (3.8%) | M 55.4%  F 44.6% | RT-PCR | F508del homozygous 57 (43.8%)  F508del heterozygous 46 (35.4%)  Other 27 (20.8%) | 40 (32.0%) | 65 (51.2%) | N/A | 82 (79.6%) | CFTR modulator  31 (24.6%)  Azitromicin  30 (39%) | 23  incidence in lung transplanted pwCF (8.43/1000, 95% CI: 5.35-12.62) vs non-lung transplanted pwCF (2.36/1000, 95% CI: 1.94-2.86) | 12 (9.2%) | 5 (3.8%)  Of these 3 had lung transplant | 75 (58.1%) | Additional iv antibiotics 49.5%  Oxygen 24/86 (27.9%)  respiratory support 12/80 (12.5%)  Of these  NIV 5 invasive ventilation 5 ECMO 2 | Incidence was higher  in pwCF < 49 y compared to the general population  Compared to the age-matched general population, pwCF have a higher incidence of SARS-CoV-2 infection and hospitalization, especially lung-transplanted pwCF |
| Jung et al (8) | Europe  ECFSPR | Prospective cohort study | 828 | Median age 24y | M 48.4%  F 51.6% | RT-PCR | F508del heterozygous  262 (31,6%)  F508del homozygous  218 (26,3%)  Others  348 (42%) | 206 (26.1%) | 346 (43,4%) | 29 (4,4%) | 660 (80.6%) | Antibiotics 38.5%  Azithromycin  38.1%  CFTR modulators 31.5%  Dornase alfa 58.3% | 78 (9,4%) | 21 (2,5%) | 11 (1,3%) | 195 (23.7%) | O2 therapy 96 (11.7%)  NIV 16 (1,9%)  Invasive ventilation12 (1,5%)  ECMO 4 (0,5%) | Update of Naerlich et al  Risk factor for severe outcome:  FEV1 <70% predicted, CFRD and lung transplants  CFTR modulator use was negatively associated with hospitalisation |
| Bain et al (9) | Argentina,Brazil,Chile, France, Germany, Italy, Russia, South Africa, Spain, Sweden,Switzerland, UK, US | Multinational retrospective cohort study | 105 | Median age 10 (IQR 6-15) | M 54%  F  46% | RT-PCR or clinical diagnosis of COVID19 | F508 del Homozygous 43/104 (41%)  F508del heterozygous 32/104 (31%)  Other 29/104(28%) | 9/100 (9%) | 31/96 (32%) | N/A | 84/100 (84%) | CFTR modulator 50/83 (60%)  Azithromycin 31/55 (56%) | 2/105  1 lung  1 liver | 1/83 (1%) | 0 | Hospitalized 24/82 (29%) | Oral antibiotics 16/43 (37%)  Iv antiobiotics: - at hospital 14/39 (36%)  -at home 4/37 (11%)  Antiviral 5/36 (14%) | Around half of children with CFRD were admitted to hospital.  Inpatients had a lower median ppFEV1 (73%) than outpatients (97%). |
| Cosgriff et al (13) | Australia, Canada, France, Ireland, Netherlands, New Zealand, UK and US  (CFRGHG) | Multinational retrospective cohort study | 40 | Median age 33 (IQR 15-57)  > | M 43%  F 57% | Positive test for SARS-CoV2 not specified which one | F508del homozygous 17 (43%) | 15 (38%) | 71% | N/A | N/A | CFTR modulators 14 (35%) | 11 | 4 (10%)  1 non trasplanted 3 transpanted | 0 | N/A | antibiotics 25 (63%) of these 10 oral and 17 IV, 2 both  oxygen supplementation 13 (33%) |  |
| McClenaghan et al (6) | Argentina, Australia, Belgium,  Brazil, Canada, Chile, France,  Germany,Ireland, Italy, Netherlands,New Zealand,Russia,  South Africa, Spain, Sweden,Switzerland, UK, US (CFRGHG) | Multicenter cohort study | 181 | Median age 27y (0-74) | M 50% F 50% | RT-PCR and/or CT scan and/or clinical diagnosis | F508del homozygous 72 (40%)  F508 del Heterozygous 65 (36%)  Other 42 (23%) | 56 (31%) | 92 (51%) | N/A | N/A | CFTR modulator 43% | 32 (18%) | 7 (25%) transplanted and 4 (4%) non trasplanted | 6:  4 non trasplanted  3 post-translanted | Non trasplanted: 66/141 (46%)  Post-transplanted: 20/27 (74%) | Supplemental O2:  Non-transplanted23/101 (23%)  Post- transplanted 12/23 (52%)  NIV: transplanted 4 (17%) vs. non transplanted 3 (3%) | Update of Cosgriff et al. |
| Moeller et al (19) | Europe | Multicenter survey | 14 | <18 years | N/A | N/A | N/A | N/A | N/A | N/A | N/A | N/A | N/A | 3 | 0 | 7 | O2 supplementation 2  Invasive ventilation 1  azithromycin 3  hydroxychloroquine 1 |  |
| Colombo et al (May 2021) (11) | Italy | Multicentre prospective matched cohort study | 16 pts  14 controls | Median age 20 y (range 0-57) | M 7 (43.8%)  F 9 (56.2%) | RT-PCR | F508del heterozygous:  6 cases  7 controls  F508del homozygous:  3 cases  1 control  Others:  7 cases  6 controls | 3 cases  5 controls | 11 cases  10 controls | N/A | 11 cases  11 controls | Antibiotics:  10 cases  8 controls  Azithromycin:  8 cases  7 controls  CS:  9 cases  10 controls  CFTR modulators:  2 cases  2 controls | 2 (12.5%) cases vs  1 (7%) control | 0 | 0 | 8 (50%) cases vs  8 (57.1%) controls | Lopinavir/ritonavir 3  Darunavir /Cobicistat 1  Hydroxy-chloroquine 3  Azithromycin 3  Other antibiotics:  11  supplemental O2 3 (18.8%) cases vs 4 (28.6%) controls  NIV 2 (12.5%) cases vs 0 controls | No significant changes in FEV1 between groups |
| Colombo et al (Dec 2021) (14) | Italy | Multicenter prospective study | 236 patients with COVID19 and CF | 81 (34,3%) <19 yrs | F 130 (55,1%)  M 106 (44,9%) | RT-PCR | F508del heterozygous:  117 (49,6%)  F508del homozygous:  53 (22,5%)  Others:  66 (28%) | 51 (21,6%) | 124 (52,5%) | 5 (2,1%) | Pancreatic insufficiency 164 (69,5%) | Antibiotics:  104  Azithromycin:  80  Steroids:  125  CFTR modulators:  54  Dornase alfa: 60 | 19 (8,1%) | 4 (1,7%) | 6 (2,5%) | 43 (18,2%) | supplemental O2 17 (7,2%)  Antivirals: 18  Hydroxychloroquine:  7  Azithromycin: 67  Other antibiotic: 99  Steroids: 77  Monoclonal antibodies: 4  Anti-thrombotic drugs: 21 | Risk factors for severe outcome:  Pancreatic insufficiency (OR 4,04), O2 therapy, FEV1<40%, underweight, diabetes, liver disease, organ transplantation  Dornase alfa associated with less severe disease (OR 0,34) |

AKI= acute kidney injury

CRD= chronic respiratory disease

CF= cystic fibrosis

CFRGHG= Cystic Fibrosis Registry Global Harmonization Group

CFRD= Cystic fibrosis related diabetes

CI= confidence interval

ECFSPR= European Cystic Fibrosis Society Patient Registry

ECMO= ExtraCorporeal Membrane Oxygenation

Iv= intravenous

NIV= non invasive ventilation

ppFEV_1_ =percentage predicted forced expiratory volume in one second

Pts=patients

pwCF= patients with cystic fibrosis

RR= risk ratio
